# Non-melanoma skin cancers and glucocorticoid therapy

**DOI:** 10.1054/bjoc.2001.1931

**Published:** 2001-09

**Authors:** M R Karagas, G L Cushing, E R Greenberg, L A Mott, S K Spencer, D W Nierenberg

**Affiliations:** Departments of Community and Family Medicine, Medicine, Pharmacology and Toxicology, and the Norris Cotton Cancer Center, Dartmouth Medical School, Lebanon, New Hampshire, 03756, USA; Mount Auburn Hospital, Cambridge, MA, 02238, USA

**Keywords:** non-melanoma skin cancer, squamous cell carcinoma, basal cell carcinoma, glucocorticoids, immunosuppressive therapy, case-control study

## Abstract

Non-melanoma skin cancer (NMSC) is an important cause of morbidity and long-term mortality in organ transplant recipients receiving immunosuppressive drugs such as azathioprine and cyclosporin, often combined with adrenocortical steroids (glucocorticoids). At lower doses, glucocorticoids alone are prescribed for other conditions including musculoskeletal, connective tissue and respiratory disorders. Presently, it is unknown whether patients taking glucocorticoids are at an increased risk of skin malignances. In a population-based case-control study in New Hampshire, USA, we compared use of glucocorticoids in 592 basal cell carcinoma (BCC) and 281 squamous cell carcinoma (SCC) cases and in 532 age and gender matched controls; neither cases nor controls had a history of organ transplantation. Participants underwent a structured personal interview regarding history of medication use and skin cancer risk factors. We used unconditional logistic regression analysis to compute odds ratios associated with glucocorticoid use for 1 month or longer while controlling for potential confounding factors. Risk of SCC was increased among users of oral glucocorticoids (adjusted odds ratio = 2.31; 95% CI = 1.27, 4.18), and risk of BCC was elevated modestly (adjusted odds ratio = 1.49; 95% CI = 0.90, 2.47). In contrast, risk of both SCC and BCC were unrelated to use of inhaled steroids. Our data suggest that use of oral glucocorticoids may increase risk of NMSC, and SCC in particular, among patients other than organ transplant recipients. We hypothesize that immunosuppression induced by oral glucocorticoids may allow these cancers to emerge from immunosurveillance. © 2001 Cancer Research Campaign http://www.bjcancer.com


*British Journal of Cancer* (2001) **85**(5), 683–686
© 2001 Cancer Research Campaign
doi: 10.1054/ bjoc.2001.1931, available online at http://www.idealibrary.com on IDEAL

http://www.bjcancer.com

### Non-melanoma skin cancers and glucocorticoid therapy

**MR Karagas[1], GL Cushing, Jr[2], ER Greenberg[1], LA Mott[1], SK Spencer[1] and DW Nierenberg[1]**

[1]Departments of Community and Family Medicine, Medicine, Pharmacology and Toxicology, and the Norris Cotton Cancer Center, Dartmouth Medical School, Lebanon, New Hampshire, 03756 USA; [2] Mount Auburn Hospital, Cambridge, MA, 02238 USA

**Summary** Non-melanoma skin cancer (NMSC) is an important cause of morbidity and long-term mortality in organ transplant recipients receiving immunosuppressive drugs such as azathioprine and cyclosporin, often combined with adrenocortical steroids (glucocorticoids). At lower doses, glucocorticoids alone are prescribed for other conditions including musculoskeletal, connective tissue and respiratory disorders. Presently, it is unknown whether patients taking glucocorticoids are at an increased risk of skin malignances. In a population-based case-control study in New Hampshire, USA, we compared use of glucocorticoids in 592 basal cell carcinoma (BCC) and 281 squamous cell carcinoma (SCC) cases and in 532 age and gender matched controls; neither cases nor controls had a history of organ transplantation. Participants underwent a structured personal interview regarding history of medication use and skin cancer risk factors. We used unconditional logistic regression analysis to compute odds ratios associated with glucocorticoid use for 1 month or longer while controlling for potential confounding factors. Risk of SCC was increased among users of oral glucocorticoids (adjusted odds ratio = 2.31; 95% CI = 1.27, 4.18), and risk of BCC was elevated modestly (adjusted odds ratio = 1.49; 95% CI = 0.90, 2.47). In contrast, risk of both SCC and BCC were unrelated to use of inhaled steroids. Our data suggest that use of oral glucocorticoids may increase risk of NMSC, and SCC in particular, among patients other than organ transplant recipients. We hypothesize that immunosuppression induced by oral glucocorticoids may allow these cancers to emerge from immunosurveillance. © 2001 Cancer Research Campaign http://www.bjcancer.com

**Keywords**: non-melanoma skin cancer; squamous cell carcinoma; basal cell carcinoma; glucocorticoids; immunosuppressive therapy; case-control study

Organ transplant recipients taking immunosuppressive drugs are at an estimated 65-fold greater risk of squamous cell carcinoma (SCC) and a 10-fold higher risk of basal cell carcinoma (BCC) (McCann, 1999). Agents used to prevent allograft rejection include cyclosporine or azathioprine, often combined with glucocorticoids. Systemic glucocorticoids alone (e.g., prednisone, prednisolone, dexamethasone and cortisone) are widely used for a variety of medical conditions including connective tissue diseases, musculoskeletal disorders and allergies. However, it is presently unknown whether these patients are at an increased risk of NMSC. Therefore, we examined the potential risks of SCC and BCC associated with glucocorticoid use in non-transplant recipients, as part of a population-based case-control study of NMSC conducted in New Hampshire, USA.

### MATERIALS AND METHODS

### Study group

To identify cases for our study, we enlisted the collaboration of dermatologists and pathology laboratories throughout New Hampshire and bordering regions (Karagas et al, 1999). We selected a random sample of BCC cases (for efficiency) and all cases of invasive SCC diagnosed from July 1, 1993 through June 30, 1995 among New Hampshire residents, aged 25–74 years. The sample of BCC cases was drawn concomitantly with the SCC cases and controls, and stratified on anatomic site, age and gender to ensure representation of the entire group of BCC diagnoses. As of March 1996, we identified 1143 potential participants. One patient was not contacted at the physician's request, 31 (3%) were reported as deceased by a household member or physician, 10 (1%) lived in households in which no one answered after 40 attempts to telephone distributed over days, evenings and weekends, 178 (16%) declined participation and 27 (2%) were deemed mentally incompetent or too ill to take part. We interviewed a total of 603 BCC and 293 SCC cases.

We chose controls from New Hampshire residents aged 25–74 years who were frequency-matched on age (25–34, 35–44, 45–54, 55–64, 65–69, 70–74 years) and gender to represent the combined distribution of the SCC and BCC cases. We selected controls (roughly equal in number to the number of BCC cases) from lists of New Hampshire residents provided by the New Hampshire Department of Transportation (for those less than 65 years old) and Health Care Financing Administration's Medicare Program (for those 65 years and older). For interviewing purposes, controls were randomly assigned reference dates comparable to the cases' diagnosis dates. Of the 820 potential controls, 12 (2%) were reported as deceased by a member of the household, for 12 (2%), no one answered in the household after 40 attempts to telephone distributed over days, evenings and weekends, 228 (28%) declined participation and 28 (3%) were deemed mentally incompetent or too ill to take part. We thus interviewed 540 controls for the study.

### Personal interview

All participants provided informed consent in accordance with the Committee for the Protection of Human Subjects at Dartmouth

*Received 15 March 2001*
*Revised 9 May 2001*
*Accepted 9 May 2001*

*Correspondence to:* MR Karagas

BJOC 01-1931 683-686 20/8/01 3:09 pm Page 684

College. Study participants completed a structured personal interview, usually at their homes. Questions included sociodemographic information (level of education), skin sensitivity to the sun after first exposure in the summer (i.e., tendency to sunburn), previous radiation treatment, use of tobacco, and time spent outdoors during working and non-working hours in the summer and the rest of the year. We asked participants if their doctor had ever prescribed corticosteroids or steroids as pills, injections or inhalers for 1 month or longer. We then asked the age they were first treated, condition for which the corticosteroids were prescribed, the name of the drug, dose, and the duration of treatment. To aid recall, we developed a list of glucocorticoid drugs (trade name, generic name and description) and a pictorial guide showing the most commonly used drugs grouped by pill colour, size and shape. To minimize potential reporting bias, we did not reveal the specific hypotheses of interest to either the interviewer or participant, and we did not inform the interviewers of the case-control status of participants.

### Statistical analysis

We computed the odds ratio (OR) and 95% confidence interval (CI) of BCC and SCC associated with use of glucocorticoids prior to the reference date using unconditional logistic regression, taking into account multiple confounding factors (Breslow and Day, 1980). In addition to age and gender, we assessed the potentially confounding effects of skin reaction to the sun (severe sunburn with blistering, painful sunburn, mild sunburn with some tanning and tanning with no sunburn), radiation treatment (no, yes), cigarette smoking history (never, former, current), and level of education (less than college, college, graduate/professional school). All relative risk estimates of SCC risk were adjusted for age, gender and sun sensitivity, and estimates of BCC for age and gender. No other factors appreciably influenced the results.

### RESULTS

A total of 592 BCC, 281 SCC and 532 controls had available information on previous glucocorticoid use and no known history of an organ transplantation (4 BCC, 8 SCC and no controls had an organ transplant). Of the remaining subjects, SCC cases were older on average than BCC cases or controls (Table 1). A higher proportion of BCC and SCC cases than controls reported a tendency to sunburn, a history of radiation treatment, and graduate or professional school education (Table 1). The overall average number of hours spent outdoors during the summer months either recreationally or occupationally was roughly similar for SCC and BCC cases and controls (Table 1).

Overall, 13% of SCC cases, 10% of BCC cases and 8% of controls reported using inhaled or oral glucocorticoids for one month or longer (Table 1). The distribution of medical indications for glucocorticoid therapy did not differ notably between cases and controls (Table 1). Women reported using glucocorticoids more often than men (12.7% of women versus 8.9% of men); however, age, sun sensitivity, time spent outdoors, and level of educational attainment varied little according to glucocorticoid use (data not shown). Prednisone accounted for more than 80% of reported use of oral glucocorticoids.

Use of oral, but not inhaled glucocorticoids, was associated with an increased risk of both SCC and BCC. For SCC, risk was elevated more than two-fold (adjusted odds ratio = 2.31; 95% CI = 1.27, 4.18; $P = 0.01$) and was stronger for current than prior use (Table 2). We did not detect a trend in SCC risk with duration of use, but relatively few subjects indicated using glucocorticoids for more than 6 months (Table 2). Risk of BCC risk was modestly, and not clearly, elevated (adjusted odds ratio = 1.49; 95% CI = 0.90, 2.47; $P = 0.12$). Cases and controls reported a similar prevalence of inhaled glucocorticoid use (adjusted odds ratio for BCC = 0.76, 95% CI = 0.39, 1.47; $P = 0.49$; adjusted odds ratio for SCC = 1.44, 95% CI = 0.68, 3.05; $P = 0.36$).

### DISCUSSION

Long-term immunosuppressive therapy to prevent allograft rejection markedly increases the occurrence of NMSCs. In organ transplant recipients, reported relative risks of NMSC range from 7 to as high as 250 (Blohme and Larko, 1984; Dyall-Smith and Ross, 1995; Gupta et al, 1986; Hartevelt et al, 1990; Hoxtell et al, 1977; Jensen et al, 1999; Kinlen, 1996; O'Connell et al, 1986; Sheiner et al, 2000). Treatment to prevent allograft rejection usually includes a multi-agent regimen of a cytotoxic drug (e.g., azathioprine), an agent affecting signal transduction in T-lymphocytes (e.g., cyclosporin), often combined with a glucocorticoid (e.g., prednisone), which suppresses T-cell proliferation and immune response. Other groups of patients use glucocorticoids alone, but at lower doses and generally for shorter durations. In our study, these individuals had an increased risk of SCC, and possibly of BCC, albeit to a lesser extent than that experienced by organ transplant recipients.

There are a few reports of NMSC among patients using immunosuppressive drugs for conditions other than organ transplantation. Kinlen and colleagues observed three cases of SCC versus 0.60 expected in a series of 1634 patients without transplants who were treated with azathioprine, cyclophosphamide, or chlorambucil (Kinlen, 1996). In a cohort of rheumatoid arthritis patients, skin cancer occurred in 19 out of 119 of the cyclophosphamide-treated patients (3 SCC cases) compared to 6 out of 119 patients not receiving cyclophosphamide (one SCC case) (Radis et al, 1995). In our study, we did not specifically ask subjects whether they took cyclophosphamide or other immunosuppressive agents. Nonetheless, our results for patients who reported taking glucocorticoids (and potentially combined with other agents), are consistent with prior studies on cyclophosphamide and other agents used in non-transplant recipients.

In contrast to the enhanced NMSC risk associated with oral glucocorticoids, we found no association between inhaled glucocorticoids and risk of either SCC or BCC. This result is not surprising, since inhaled glucocorticoids have limited systemic effects (Hardman et al, 1996). The fact that cases and controls were about equally likely to report using inhaled glucocorticoids suggests that NMSC cases do not report more use of drugs in general, or that increased surveillance for skin cancer among patients with chronic diseases can explain the observed association between oral glucocorticoids and NMSC. We did not note any particular condition for which NMSC patients reported taking glucocorticoids more than controls; however, the number of cases in each category was small.

The excess incidence of NMSCs seen in organ transplant recipients is likely due to the immunodeficient state itself. In a cohort study of 1098 renal transplant recipients, the excess NMSC risk occurred irrespective of the type of immunosuppressive therapy used (Bouwes Bavinck et al, 1996). Further, in a report of 2562

 © 2001 Cancer Research Campaign

BJOC 01-1931 683-686 20/8/01 3:09 pm Page 685

**Table 1** Selected characteristics of basal cell carcinoma and squamous cell carcinoma cases and controls

| Variable* | Controls *n* = 532 (%) | BCC cases *n* = 592 (%) | SCC cases *n* = 281 (%) |
|---|---|---|---|
| Gender | | | |
| Male | 319 (60.0) | 336 (56.8) | 177 (63.0) |
| Female | 213 (40.0) | 256 (43.2) | 104 (37.0) |
| Age (years) | | | |
| <60 | 202 (38.0) | 268 (45.3) | 64 (22.8) |
| 60–64 | 70 (13.2) | 86 (14.5) | 42 (15.0) |
| 65–69 | 137 (25.8) | 118 (19.9) | 71 (25.3) |
| 70–74 | 123 (23.1) | 120 (20.3) | 104 (37.0) |
| Skin reaction to sun exposure for 1 hour the first time in summer | | | |
| Severe sunburn with blistering | 35 (6.6) | 26 (4.4) | 30 (10.7) |
| Painful sunburn followed by peeling | 135 (25.5) | 212 (35.9) | 99 (35.4) |
| Mildly burnt with some tanning | 264 (49.9) | 307 (52.0) | 126 (45.0) |
| Tan without sunburn | 95 (18.0) | 46 (7.8) | 25 (8.9) |
| Mean (SD) lifetime hours/week outdoors in summer | | | |
| Recreational | 14.9 (6.8) | 14.8 (6.6) | 14.4 (6.5) |
| Occupational | 10.2 (8.6) | 9.3 (8.9) | 10.5 (9.8) |
| Radiation treatment | | | |
| No | 492 (92.5) | 514 (86.8) | 245 (87.5) |
| Yes | 40 (7.5) | 78 (13.2) | 35 (12.5) |
| Smoking status | | | |
| Never | 177 (33.3) | 254 (42.9) | 89 (31.7) |
| Former | 251 (47.3) | 251 (42.4) | 142 (50.5) |
| Current | 103 (19.4) | 87 (14.7) | 50 (17.8) |
| Education | | | |
| High school or technical school | 258 (48.1) | 229 (38.7) | 122 (43.4) |
| College | 169 (31.8) | 215 (36.3) | 89 (31.7) |
| Graduate or professional school | 107 (20.1) | 148 (25.0) | 70 (24.9) |
| Any steroid use (inhaled or oral) | | | |
| None | 491 (92.3) | 534 (90.2) | 245 (87.2) |
| Oral only | 21 (4.0) | 40 (6.8) | 23 (8.2) |
| Inhaled only | 15 (2.8) | 15 (2.5) | 10 (3.6) |
| Oral and Inhaled | 5 (0.9) | 3 (0.5) | 3 (1.1) |
| Reason for oral steroid use (users only)† | | | |
| Respiratory conditions and asthma | 9 (29.0) | 8 (18.2) | 9 (33.3) |
| Musculoskeletal and connective tissue disease | 6 (19.4) | 12 (27.3) | 8 (29.6) |
| Neoplasm | 1 (3.2) | 4 (9.1) | 2 (7.4) |
| Allergy | 7 (22.6) | 6 (13.6) | 1 (3.7) |
| Gastrointestinal disease | 1 (3.2) | 5 (11.4) | 2 (7.4) |
| Other | 7 (22.6) | 9 (20.5) | 5 (18.5) |

*Five individuals had missing data on skin reaction to sun exposure (1 BCC, 1 SCC, and 3 controls); one SCC case had missing data on radiation treatment; one control had missing data on smoking status; two individuals (1 BCC and 1 SCC) had missing data on indication for steroid use. †Four cases (2 BCC and 2 SCC) were treated for conditions in more than one category.

heart and kidney recipients, the magnitude of the NMSC risk related to the degree of drug-induced immunosuppression (Jensen et al, 1999). In animal experiments, immunosuppression produced by exposure to ultraviolet light plays a mechanistic role in skin carcinogenesis (Kripke, 1994).

Physicians prescribe glucocorticoids for patients with a variety of medical conditions because of their immunosuppressive and anti-inflammatory effects (Hardman et al, 1996), and in our study, about 8% of population controls reported using glucocorticoids for one month or longer. NMSC is a frequent and growing problem in Caucasian populations (Coebergh et al, 1991; Gallagher et al, 1990; Kaldor et al, 1993; Karagas et al, 1999; Levi et al, 1995; Staples et al, 1998). Our data along with others require further confirmation and indicate that glucocorticoids may contribute to NMSC occurrence.

### ACKNOWLEDGEMENTS

The authors are indebted to the collaboration of the New Hampshire Skin Cancer Study Group. The following physicians are members: Duane R Anderson, MD; Robert W Averill, MD; Anthony J Aversa, MD; Bruce A Bairstow, MD; Richard D Baughman, MD; Lawrence G Blasik, MD; James Campbell, MD; Carolyn Carroll, MD; William E Clendenning, MD; Daniel W Collison, MD; Jorge L Crespo, MD; Frederick W Danby, MD; Stephen M Del Guidice, MD; Robert L Dimond, MD; Wilmot S Draper, MD; Jeremy P Finkle, MD; William E Frank, MD; John L Fromer, MD; Norman C Goldberg, MD; David Goldminz, MD; Robert Gordon, MD; David S Greenstein, MD; Thomas P Habif, MD; Charles Hammer, MD; Tom Hokanson, PA; Steve A Joselow, MD; Michael D Lichter, MD; Maritza O Liranzo, MD; Lynette

© 2001 Cancer Research Campaign

BJOC 01-1931 683-686 20/8/01 3:09 pm Page 686

**Table 2** Odds ratios (95% confidence intervals) for oral glucocorticoid use and basal cell carcinoma and squamous cell carcinoma*

| Variable | Controls Number (%) | Basal cell carcinoma Number (%) | Basal cell carcinoma Adjusted odds ratio (95% CI)† | Squamous cell carcinoma Number (%) | Squamous cell carcinoma Adjusted odds ratio (95% CI)‡ |
|---|---|---|---|---|---|
| Any oral use | | | | | |
| No steroid use | 491 (95.0) | 534 (92.6) | 1.00 – | 245 (90.4) | 1.00 – |
| Yes | 26 (5.0) | 43 (7.5) | 1.49 (0.90, 2.47) | 26 (9.6) | 2.31 (1.27, 4.18) |
| Current or past use of oral glucocorticoid use§ | | | | | |
| No steroid use | 491 (95.2) | 534 (92.7) | 1.00 – | 245 (90.4) | 1.00 – |
| Current use | 10 (1.9) | 18 (3.1) | 1.66 (0.76, 3.64) | 15 (5.5) | 3.67 (1.57, 8.63) |
| Past use | 15 (2.9) | 24 (4.2) | 1.42 (0.73, 2.74) | 11 (4.1) | 1.59 (0.69, 3.68) |
| Total duration of oral glucocorticoid use§ | | | | | |
| No steroid use | 491 (95.2) | 534 (92.7) | 1.00 – | 245 (90.4) | 1.00 – |
| ≤ 6 months | 17 (3.3) | 24 (4.2) | 1.13 (0.53, 2.43) | 17 (6.3) | 2.45 (1.05, 5.72) |
| 7 months–3 years | 5 (1.0) | 11 (1.9) | 1.70 (0.71, 4.06) | 4 (1.5) | 2.26 (0.78, 6.56) |
| > 3 years | 3 (0.6) | 7 (1.2) | 2.15 (0.74, 6.25) | 5 (1.9) | 2.52 (0.73, 8.69) |

*Excludes individuals who used only inhaled steroids. †Adjusted for age and sex. ‡Adjusted for age and sex and skin reaction to sun exposure. §One control and one BCC have missing data.

Margesson, MD; Michael A Mittleman, MD; Jose Peraza, MD; Robert B Posnick, MD; Warren M Pringle, MD; Mark Quitadamo, MD; Pauline B Reohr, MD; N. Chester Reynolds, MD; Anna Ryan, MD; Peter Sands, MD; Mitchell E Schwartz, MD; Gregory Seymour, MD; Steven K Spencer, MD; James C Starke, MD; Susan Sullivan, MD; N. Hakan Thyresson, MD; Andrew P Truhan, MD; Mauray J Tye, MD; John Watson, MD, K. William Waterson, MD; Robert Willer, MD; Kathryn Zug, MD. The authors thank Dr Katherine Carlson for assistance in the development of the questionnaire and photo aids and Ms Virginia Stannard for coordination of the study. The study was funded by the USA National Institutes of Health grant NCI CA 57495 and was cosponsored by the New Hampshire Society of Dermatology.

### REFERENCES

Blohme I and Larko O (1984) Premalignant and malignant skin lesions in renal transplant patients. *Transplantation* **37**: 165–167

Bouwes Bavinck JN, Hardie DR, Green A, Cutmore S, MacNaught A, O'Sullivan B, Siskind V, Van Der Woude FJ and Hardie IR (1996) The risk of skin cancer in renal transplant recipients in Queensland, Australia. A follow-up study. *Transplantation* **61**: 715–721

Breslow N and Day N (1980) *Statistical Methods in Cancer Research. Volume I – The Analysis of Case-Control Studies*. IARC: Lyon

Coebergh JW, Neumann HA, Vrints LW, van der Heijden L, Meijer WJ and Verhagen-Teulings MT (1991) Trends in the incidence of non-melanoma skin cancer in the SE Netherlands 1975–1988: a registry-based study. *Br J Dermatol* **125**: 353–359

Dyall-Smith D and Ross JB (1995) Cutaneous malignancies in renal transplant recipients from Nova Scotia, Canada. *Australas Dermatol* **36**: 79–82

Gallagher RP, Ma B, McLean DI, Yang CP, Ho V, Carruthers JA and Warshawski LM (1990) Trends in basal cell carcinoma, squamous cell carcinoma, and melanoma of the skin from 1973 through 1987. *J Am Acad Dermatol* **23**: 413–421

Gupta AK, Cardella CJ and Haberman HF (1986) Cutaneous malignant neoplasms in patients with renal transplants. *Arch Dermatol* **122**: 1288–1293

Hardman JG, Gilman AG and Limbird LE (1996) *Goodman's & Gilman's The Pharmacological Basis of Therapeutics*. McGraw-Hill: New York

Hartevelt MM, Bavinck JN, Kootte AM, Vermeer BJ and Vandenbroucke JP (1990) Incidence of skin cancer after renal transplantation in The Netherlands. *Transplantation* **49**: 506–509

Hoxtell EO, Mandel JS, Murray SS, Schuman LM and Goltz RW (1977) Incidence of skin carcinoma after renal transplantation. *Arch Dermatol* **113**: 436–438

Jensen P, Hansen S, Moller B, Leivestad T, Pfeffer P, Geiran O, Fauchald P and Simonsen S (1999) Skin cancer in kidney and heart transplant recipients and different long-term immunosuppressive therapy regimens. *J Am Acad Dermatol* **40**: 177–186

Kaldor J, Shugg D, Young B, Dwyer T and Wang YG (1993) Non-melanoma skin cancer: Ten years of cancer-registry-based surveillance. *Int J Cancer* **53**: 886–891

Karagas MR, Greenberg ER, Spencer SK, Stukel TA and Mott LA (1999) Increase in incidence rates of basal cell and squamous cell skin cancer in New Hampshire, USA. *Int J Cancer* **81**: 555–559

Kinlen L (1996) Immunologic factors, including AIDS. In: *Cancer Epidemiology and Prevention*, Schottenfeld D and Fraumeni JJ (eds) pp 532–545. Oxford University Press: New York

Kripke ML (1994) Ultraviolet radiation and immunology: something new under the sun – presidential address. *Cancer Res* **54**: 6102–6105

Levi F, Franceschi S, Te VC, Randimbison L and La Vecchia C (1995) Trends of skin cancer in the Canton of Vaud, 1976–92. *Br J Cancer* **72**: 1047–1053

McCann J (1999) Can skin cancers be minimized or prevented in organ transplant recipients? *J Natl Cancer Inst* **91**: 911–913

O'Connell BM, Abel EA, Nickoloff BJ, Bell BJ, Hunt SA, Theodore J, Shumway NE and Jacobs PH (1986) Dermatologic complications following heart transplantation. *J Heart Transplant* **5**: 430–436

Radis CD, Kahl LE, Baker GL, Wasko MCM, Cash JM, Gallatin S, Stolzer BL, Agarwal AK, Medsger TA and Kwoh CK (1995) Effects of cyclophosphamide on the development of malignancy and on long-term survival of patients with rheumatoid arthritis: a 20-year followup study. *Arthritis Rheum* **38**: 1120–1127

Sheiner PA, Magliocca JF, Bodian CA, Kim-Schluger L, Altaca G, Guarrera JV, Emre S, Fishbein TM, Guy SR, Schwartz ME and Miller CM (2000) Long-term medical complications in patients surviving > or = 5 years after liver transplant. *Transplantation* **69**: 781–789

Staples M, Marks R and Giles G (1998) Trends in the incidence of non-melanocytic skin cancer (NMSC) treated in Australia 1985–1995: are primary prevention programs starting to have an effect? *Int J Cancer* **78**: 144–148

 *© 2001 Cancer Research Campaign*

## References

[PDF_00299] Blohmé I., Larkö O. (1984). Premalignant and malignant skin lesions in renal transplant patients.. Transplantation.

[PDF_00301] Bouwes Bavinck J. N., Hardie D. R., Green A., Cutmore S., MacNaught A., O'Sullivan B., Siskind V., Van Der Woude F. J., Hardie I. R. (1996). The risk of skin cancer in renal transplant recipients in Queensland, Australia. A follow-up study.. Transplantation.

[PDF_00307] Coebergh J. W., Neumann H. A., Vrints L. W., van der Heijden L., Meijer W. J., Verhagen-Teulings M. T. (1991). Trends in the incidence of non-melanoma skin cancer in the SE Netherlands 1975-1988: a registry-based study.. Br J Dermatol.

[PDF_00311] Dyall-Smith D., Ross J. B. (1995). Cutaneous malignancies in renal transplant recipients from Nova Scotia, Canada.. Australas J Dermatol.

[PDF_00313] Gallagher R. P., Ma B., McLean D. I., Yang C. P., Ho V., Carruthers J. A., Warshawski L. M. (1990). Trends in basal cell carcinoma, squamous cell carcinoma, and melanoma of the skin from 1973 through 1987.. J Am Acad Dermatol.

[PDF_00317] Gupta A. K., Cardella C. J., Haberman H. F. (1986). Cutaneous malignant neoplasms in patients with renal transplants.. Arch Dermatol.

[PDF_00324] Hoxtell E. O., Mandel J. S., Murray S. S., Schuman L. M., Goltz R. W. (1977). Incidence of skin carcinoma after renal transplantation.. Arch Dermatol.

[PDF_00326] Jensen P., Hansen S., Møller B., Leivestad T., Pfeffer P., Geiran O., Fauchald P., Simonsen S. (1999). Skin cancer in kidney and heart transplant recipients and different long-term immunosuppressive therapy regimens.. J Am Acad Dermatol.

[PDF_00330] Kaldor J., Shugg D., Young B., Dwyer T., Wang Y. G. (1993). Non-melanoma skin cancer: ten years of cancer-registry-based surveillance.. Int J Cancer.

[PDF_00333] Karagas M. R., Greenberg E. R., Spencer S. K., Stukel T. A., Mott L. A. (1999). Increase in incidence rates of basal cell and squamous cell skin cancer in New Hampshire, USA. New Hampshire Skin Cancer Study Group.. Int J Cancer.

[PDF_00339] Kripke M. L. (1994). Ultraviolet radiation and immunology: something new under the sun--presidential address.. Cancer Res.

[PDF_00341] Levi F., Franceschi S., Te V. C., Randimbison L., La Vecchia C. (1995). Trends of skin cancer in the Canton of Vaud, 1976-92.. Br J Cancer.

[PDF_00343] McCann J. (1999). Can skin cancers be minimized or prevented in organ transplant patients?. J Natl Cancer Inst.

[PDF_00345] O'Connell B. M., Abel E. A., Nickoloff B. J., Bell B. J., Hunt S. A., Theodore J., Shumway N. E., Jacobs P. H. (1986). Dermatologic complications following heart transplantation.. J Heart Transplant.

[PDF_00348] Radis C. D., Kahl L. E., Baker G. L., Wasko M. C., Cash J. M., Gallatin A., Stolzer B. L., Agarwal A. K., Medsger T. A., Kwoh C. K. (1995). Effects of cyclophosphamide on the development of malignancy and on long-term survival of patients with rheumatoid arthritis. A 20-year followup study.. Arthritis Rheum.

[PDF_00352] Sheiner P. A., Magliocca J. F., Bodian C. A., Kim-Schluger L., Altaca G., Guarrera J. V., Emre S., Fishbein T. M., Guy S. R., Schwartz M. E. (2000). Long-term medical complications in patients surviving > or = 5 years after liver transplant.. Transplantation.

[PDF_00356] Staples M., Marks R., Giles G. (1998). Trends in the incidence of non-melanocytic skin cancer (NMSC) treated in Australia 1985-1995: are primary prevention programs starting to have an effect?. Int J Cancer.

